# Utilization of leukapheresis and CD4 positive selection in Treg isolation and the *ex-vivo* expansion for a clinical application in transplantation and autoimmune disorders

**DOI:** 10.18632/oncotarget.13101

**Published:** 2016-11-04

**Authors:** Karolina Gołąb, Randall Grose, Piotr Trzonkowski, Amittha Wickrema, Martin Tibudan, Natalia Marek-Trzonkowska, Sabrina Matosz, Julia Solomina, Diane Ostrega, J. Michael Millis, Piotr Witkowski

**Affiliations:** ^1^ Department of Surgery, Section of Transplantation, University of Chicago, Chicago, USA; ^2^ South Australian Health and Medical Research Institute, University of Adelaide, Australia; ^3^ Department of Clinical Immunology and Transplantology, Medical University of Gdansk, Gdansk, Poland; ^4^ Department of Medicine, Section of Hematology-Oncology, Cancer Research Center, University of Chicago, Chicago, USA; ^5^ Department of Family Medicine, Medical University of Gdansk, Gdansk, Poland

**Keywords:** regulatory T cells, clinical application, leukapheresis, CD4+ cells, immunosupressive therapy

## Abstract

Adoptive transfer of T regulatory cells (Tregs) is of great interest as a novel immunosuppressive therapy in autoimmune disorders and transplantation. Obtaining a sufficient number of stable and functional Tregs generated according to current Good Manufacturing Practice (cGMP) requirements has been a major challenge in introducing Tregs as a clinical therapy. Here, we present a protocol involving leukapheresis and CD4^+^ cell pre-enrichment prior to Treg sorting, which allows a sufficient number of Tregs for a clinical application to be obtained. With this method there is a decreased requirement for *ex-viv*o expansion. The protocol was validated in cGMP conditions. Our final Treg product passed all release criteria set for clinical applications. Moreover, during expansion Tregs presented their stable phenotype: percentage of CD4^+^CD25^hi^CD127^−^ and CD4^+^FoxP3^+^ Tregs was > 95% and > 80%, respectively, and Tregs maintained proper immune suppressive function *in vitro*. Our results suggest that utilization of leukapheresis and CD4 positive selection during Treg isolation improves the likelihood of obtaining a sufficient number of high quality Treg cells during subsequent *ex-vivo* expansion and they can be applied clinically.

## INTRODUCTION

T regulatory cells (Tregs) have ability to regulate immune reaction and maintain self-tolerance attenuating autoimmunity, allergy, pathogen-induced immunopathology and over-exuberant immune responses [1–4]. Due to their properties, Tregs are favorable for therapeutic tools in tolerance restoration/induction in autoimmunity disorders and in cell/solid organ transplantation, however a high number of cells are required to obtain clinical effects [5]. Therefore, the strategy for clinical applications of the Tregs involves their isolation and *ex vivo* expansion followed by the infusion into a patient. After successful Treg application in pre-clinical models, first clinical trials with Tregs were conducted in graft-versus-host disease (GvHD) after bone marrow transplantation [6–9] as well as in new onset of type 1 Diabetes Mellitus (T1DM) [10–12]. Several new clinical trials with Tregs are ongoing in solid organ transplantation and other immune disorders [13, 14].

Nevertheless many aspects of clinical application of Tregs as immunotherapy still remain challenging. Tregs are not a homogenous cell population and at least two main subpopulations are distinguished: natural, thymic Tregs (tTregs) and induced or adaptive Tregs (aTregs) [15, 16]. tTregs are generated in the thymus and express high levels of CD25 and lineage marker FoxP3 (forkhead transcription factor box P3) [17], whereas aTregs are generated in periphery upon contact with antigen from CD4^+^CD25^−^ precursors [18]. Moreover, most of the single surface markers that are used for defining Tregs (like FoxP3) are not exclusively Treg-specific and upon certain conditions can be expressed on activated conventional Th cells [1]. That complicates Treg identification during the isolation and quality check of the final product before clinical application. In our work, we focused on *ex vivo* expansion of polyclonal tTregs, which exhibit *in vivo* and *in vitro* stable regulatory function. Those Tregs can be identified as cells with the phenotype: CD4^+^CD25^high^CD127^−^FoxP3^+^ and are also characterized by stable methylation status of TSDR (Treg-Specific Demethylated Region) in the FoxP3 gene [19–21]. Isolation of Tregs can be done via immunomagnetic bead separation or fluorescence-activated cell sorting (FACS), or by combination of both these methods. Immunomagnetic isolation, despite being more robust, is less accurate and does not provide the ability to select as pure and as precisely defined Treg population as the sorting technique [22, 23].

Most approaches utilize the patient's own peripheral blood as a source of Tregs. Alternatively, umbilical cord blood (UCB) could be used, but so far a minority of the patients have banked UCB. Isolated Tregs need to be expanded *in vitro* due to low number and frequency in the blood. Treg proliferation is usually induced by T-cell receptor (TCR) stimulation. Microbeads covered with anti-CD3/CD28 antibodies can be used for such stimulation after which Tregs are expanded in the presence of recombinant human interleukin-2 (IL-2) [6, 10, 23, 24]. The number of Treg cells that could be obtained after expansion often varies. Donor age is only one example of many factors which can affect fold of expansion [10, 25, 26]. Even despite optimized expansion protocol (culture conditions and time), lower starting with numbers of Tregs requires higher fold increase during expansion to generate sufficient final number of Tregs for clinical application, which leads to higher risk of failure [26]. As increasing blood drawing volume to obtain higher starting Treg number is impractical, we decided to utilize leukapheresis for initial Treg cell retrieval. Such modification allowed isolating much higher Treg numbers limiting the risk of achieving insufficient Treg final numbers after the expansion. Leukapheresis product has been tested previously in other protocols for Treg isolation. However, in these protocols Tregs were not subsequent expanded or isolated via immunomagnetic beads separation [27, 28]. In our protocol, we utilized leukapheresis in combination with CD4^+^ positive magnetic selection (pre-enrichment) and FACS sorting for enhanced purity and number of Tregs.

To utilize Treg-based therapy in clinical settings, cell processing must be performed according to current Good Manufacturing Practice (cGMP) guidelines, which ensures product quality, limiting the risk of cross-contamination and pathogenic transmission [29]. Additionally, as the United States law (21 CFR § 1271) states, the Food and Drug Administration (FDA) requires cell-based product to be tested and documented for sterility, identity, purity and potency before it can be administered into a patient. That requires passing an array of tests meeting final release criteria before the final Treg product can be safely released for a clinical use.

Here, we present validation of our Treg production protocol that we established according to cGMP guidelines and FDA requirements for cell-based products. Before establishing the final clinical-grade protocol, we performed several research-grade Treg isolations/expansions to optimize and ensure the best conditions for Treg processing [30]. After that, to check the feasibility of our established protocol, we performed several more pre-clinical grade Treg isolations and expansions, and we present the results here. In the final step, we validated the protocol in the cGMP settings with the use of all clinical-grade reagents. All together, we show below that we can provide in consistent manner, sterile, well-defined Treg cell final product to be considered by FDA for Investigational New Drug (IND) approval and to be tested for safety and effectiveness in clinical studies.

## RESULTS

### Pre-clinical grade Treg isolations and expansions

**Figure 1:**
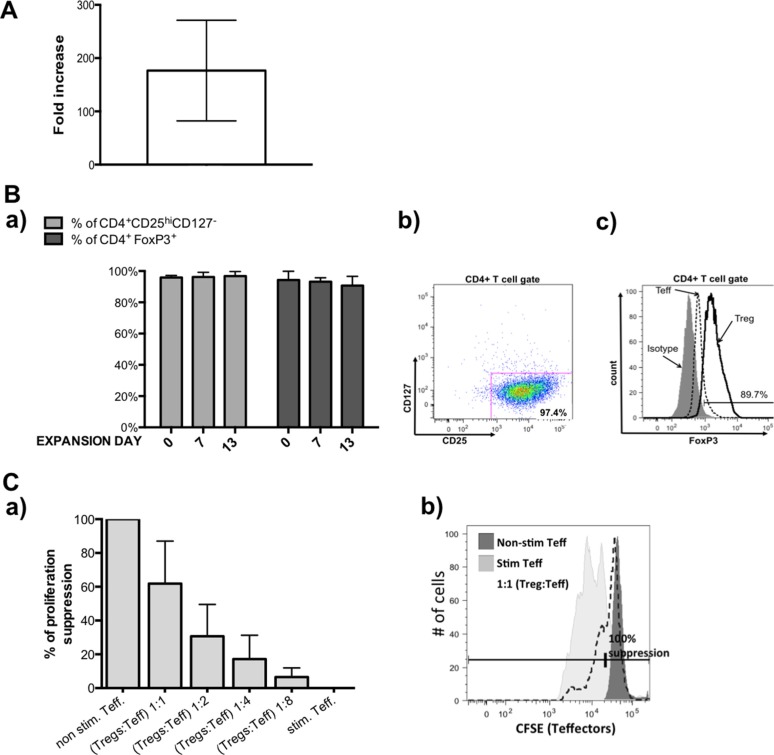
Results from pre-clinical grade Treg expansions (**A**) Fold increase in Treg cell number achieved during pre-validation expansions. (**B**) Treg phenotype during expansion, a) % of CD4^+^CD25^hi^CD127^lo/−^(light gray bars) and % CD4^+^FoxP3^+^ (dark gray bars) within expanded Tregs was maintained on high level, b) representative flow cytometric dot plot showing frequency of CD4^+^CD25^hi^CD127^lo/−^ cells in analyzed sample of Tregs, c) representative flow cytometric histogram overlay showing analysis of FoxP3 expression in sample of Tregs (black line), expanded on the same time Teffectors that served as the control (dotted line) and isotype (on gray) based on which gate was placed. (**C**) Suppressive abilities of expanded Tregs in *in vitro* assay. (a) Expanded Tregs indicated good suppressive abilities, (b) representative of flow cytometric histogram showing CFSE signal of stained Teffectors; non-stimulated with anti-CD3/CD28 beads Teffectors (dark gray) served as control for 100% suppression of proliferation and % of suppression in conditions with Tregs were calculated using following formula: (% of suppressed cells in Teff. co-cultured with Tregs × 100) / % of suppressed cells in non-stimulated Teff. Results throughout the figure are expressed as mean ± SD; *n* ≥ 3.

For Treg isolations we used leukapheresis product obtained from healthy donors that contained according to the provider, 6 × 10^9^ – 10 × 10^9^ peripheral blood mononuclear cells (PBMCs). Our phenotype analysis showed that in PBMCs, 35 ± 9.3% cells were CD4 positive and 5.8 ± 0.3% of those cells expressed Treg markers (CD4^+^CD25^hi^CD127^−/lo^). After immunomagnetic CD4^+^ cells isolation on CliniMACS^®^ device, we achieved 1.9 × 10^9^ ± 7.8 × 10^8^ CD4^+^ cells with purity of 99.2 ± 0.3%, viability of 93 ± 8.4 % and containing 6 ± 0.1% Tregs (CD4^+^CD25^hi^CD127^−/lo^ cells). Only a portion of those cells were used for further processing, whereas remaining parts were cryopreserved for future use. Similarly, only a part of subsequently isolated CD4^+^ cells were sorted thus obtaining pure Tregs, which were next expanded, according to described below clinical-grade 13 day expansion protocol with average 176.7 fold increase in Treg number (Figure 1A). During expansion, Tregs maintained stable phenotype - % of cells with expression CD4^+^CD25^hi^CD127^−/lo^ was > 95% and with CD4^+^FoxP3^+^ > 85% at all expansion checkpoints (Figure 1B). Expanded Tregs also demonstrated good suppressive abilities in *in vitro* suppression of proliferation assay (Figure 1C).

### Validation of clinical-grade Treg isolation and expansion protocol

**Figure 2:**
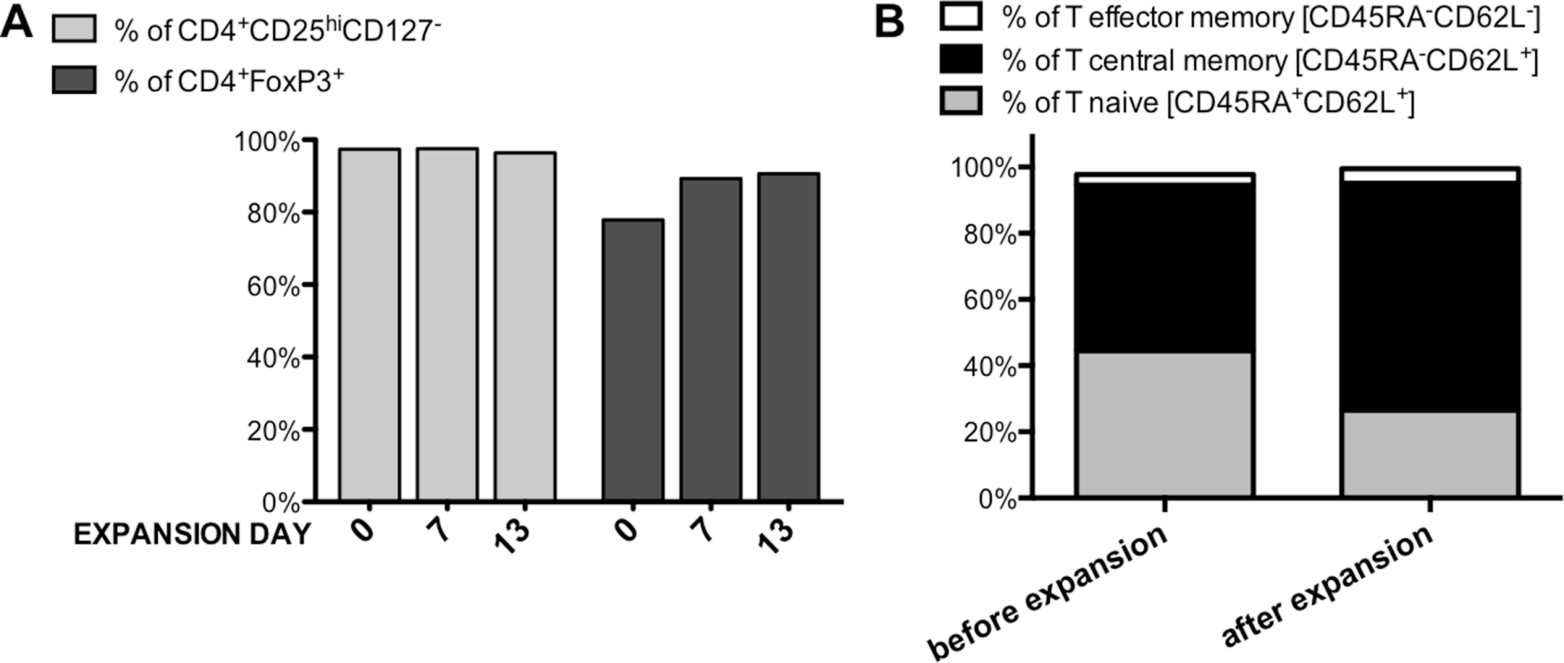
Treg Phenotype during clinical-grade Treg isolation and expansion process (**A**) Isolated and expanded Tregs presented stable CD4^+^CD25^hi^CD127^lo/−^ phenotype (light gray bars) together with maintained high expression of transcription factor FoxP3 (dark gray bars) during all Quality Check Points, (**B**) The percentage of naïve, central memory and effector memory cells in Tregs before and after 13-day expansion; *n* = 1.

For clinical-grade validation of Treg isolation and expansion protocol we obtained leukapheresis product from the same provider. Accordingly to our phenotype analysis 55.2% of PBMCs were CD4^+^ positive and 8% of them were CD4^+^CD25^hi^CD127^−/lo^. CD4^+^ cells were isolated in the number of 1.6 × 10^9^ with the purity of 98.9%, viability of 94% and they contained 8.1% Tregs (CD4^+^CD25^hi^CD127^−/lo^ cells). Sorting of 5 × 10^8^ of CD4^+^ cells allowed us to obtain 9.7 × 10^6^ of Tregs with the purity 97.4% and 77.9% measured by % of cells expressing CD4^+^CD25^hi^CD127^−/lo^ and CD4^+^FoxP3^+^, respectively (Figure 2A). Sorted Tregs were characterized by good viability (88.6%) and sterility based on negative fungal and bacterial growth in the extracted sample. After expansion of isolated Tregs stimulated with anti-CD3/anti-CD28-coated beads during 13 day *in vitro* culture in medium supplemented with IL-2 we obtained 1.77 × 10^9^ Treg cells. During bead removal process on ClinExVivo^™^ MPC^®^ magnet, we observed 10% loss in Treg cell number, giving 1.6 × 10^9^ cells in the final Treg product.

### Monitoring of Treg phenotype as a quality control of Treg expansion

For quality control, we checked if the isolation and expansion process had affected the phenotype of Treg cell population. We measured and compared specific markers expression (CD4, CD25, CD127, FoxP3) just after Treg sorting- day 0, before re-stimulation at day 7, and at the end of the expansion- on day 13. Isolated Tregs maintained their specific phenotype at a stable level during expansion (> 96% of cells were CD4^+^CD25^hi^CD127^lo/−^ at all above listed time points (Figure 2A). Transcription factor FoxP3 was also expressed at high level in isolated/expanded Tregs – % of CD4^+^FoxP3^+^ Tregs was 77.9% just after isolation, 89.3% and 90.6% at day 7 and 13, respectively (Figure 2A). Such characteristic of FoxP3 expression was unique for Tregs and distinct from Tefector cells, which were analyzed in parallel for the same marker expression and served as the control (Figure 1B(c)).

It was suggested that naïve Tregs expressing CD45RA and CD62L markers are better candidates for expansion due to their stability and increased suppressive abilities in comparison to memory Treg subsets [24, 25, 31–33]. Therefore, we also analyzed frequency of T naïve CD45RA^+^CD62L^+^, T central memory CD45RA^−^CD62L^+^ and T effector memory CD45RA^−^CD62L^−^ subsets within sorted and expanded Tregs.

Before expansion, 44.5% Tregs expressed CD45RA^+^CD62L^+^ and 50.1% CD45RA^−^CD62L^+^ (Figure 1B). Over the time of expansion, CD45RA expression on Tregs decreased and proportion of naïve and central memory subsets changed to 26.5% and 68.6%, respectively (Figure 2B). However, the frequency of T effector memory CD45RA^−^CD62L^−^ subset that is considered to contain “non-suppressive” Tregs [33] was low before and after the expansion (Figure 2B).

### Expanded Tregs have ability to suppress T cell proliferation *in vitro*

**Figure 3:**
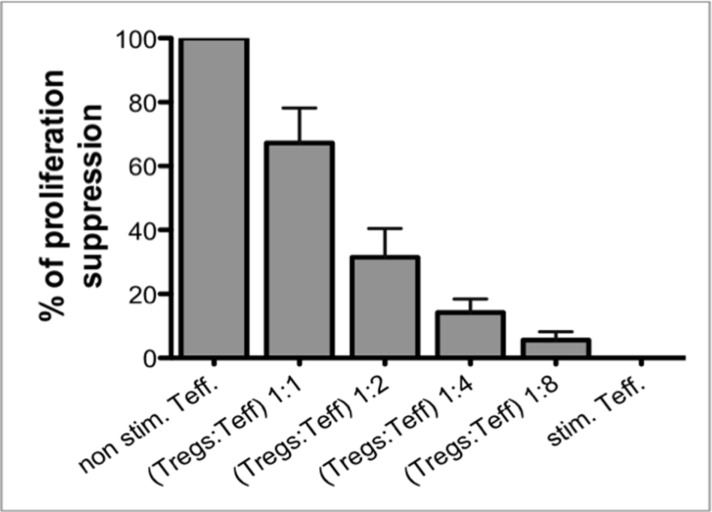
Suppression of proliferation assay as a post-expansion Treg functionality test (**A**) Expanded Tregs in the Final Product were able to suppress proliferation of CFSE-stained Teffectors (Teff) in a dose-dependent manner. Results are expressed as mean ± SD; *n* = 3.

Functionality and potency of expanded Tregs was tested in *in vitro* suppression of proliferation assay. This assay was performed at the end of expansion and served for information only. As shown in Figure 3, expanded Tregs in the final product provided the ability to suppress proliferation of Teffector cells in a dose-dependent manner.

### *Ex vivo* expanded Tregs prepared as a final Treg product fulfilled all release criteria

As required by FDA, biological product should be characterized with appropriate tests for the identity, purity, safety and potency. Those measures and acceptance values for the release criteria of the product should be established prior to clinical application [29]. Additionally, all these tests need to be validated to assure accuracy, precision, specificity, suitability and robustness with identification of criteria for the test acceptance, mean numerical limits, ranges or other attributes/variables [29].

Table 1 presents all release criteria that we established for the final Treg product in our protocol. Values for acceptance and results from the validation expansion are depicted as well. All tests used for release criteria assessment were selected accordingly to the FDA recommendation and performed by College of American Pathologists (CAP)- certified laboratories that routinely performed validations of assays they utilize. Our final Treg product manufactured according to our established protocol fulfilled all the release criteria. Details and numerical values are shown in Table 1.

**Table 1:** Release criteria of final Treg product and result of Treg processing validation

RELEASE CRITERIA	MINIMUM RELEASE CRITERIA (SPECIFICATION)	METHOD	RESULT FROM VALIDATION
**Sterility**	“no aerobic, anaeorbic and fungal growth”	CAP-certified Clinical Microbiology Laboratory	**“no aerobic,anaeorbic and fungal growth”**
**Endotoxin**	< 5 EU/kg	EndoSafe PTS Endotoxin System; calculated for an average 70kg person receiving final product	**1.28 EU/kg**
**Mycoplasma**	<0.8	Lonza MycoAlert Mycoplasma Assay	**0.399**
**Determination of Residual Beads**	< 100 beads per 3×10^6^ cells	counted on hemocytometer after cell permeabilization	**91 beads per 3 ×10^6^ cells**
**% of CD4+ cells**	>90%	flow cytometry in CAP-certified Clinical Hematology Laboratory	**99.14%**
**% of FoxP3+ cells**	>60%	flow cytometry in CAP-certified Clinical Hematology Laboratory	**90.64%**
**% of CD8+ cells**	<5%	flow cytometry in CAP-certified Clinical Hematology Laboratory	**0.35%**
**Viability**	>75%	flow cytometry in CAP-certified Clinical Hematology Laboratory	**97.79%**

## DISCUSSION

Tregs for clinical application need to be manufactured according to a protocol that follows cGMP guidelines and FDA requirements. Additionally, each step of the procedure needs to be optimized for the successful final outcome- allowing to obtain high quality and sufficient quantity, well defined, viable, functional and sterile biological cell product. In our clinical-grade Treg manufacturing protocol we perform leukapheresis instead of drawing a unit of blood [6, 10, 11] or utilizing banked blood from umbilical cord (UCB) [8, 9]. Such approach allows for starting with much higher initial number of cells for processing compared to other mentioned initial products. One unit of whole blood or UCB can yield in average 4–7.5× 10^6^ Tregs whereas over 10–20 times more Tregs- (10^8^) can be found in the product of leukapheresis [34–36]. The other advantage is, that during leukapheresis only white blood cells are collected and erythrocytes are returned to donor's circulation instead of being wasted. It is a feasible procedure, routinely performed in hospital blood bank units. During our validation processing, (even though we utilized only a small fraction (1/6–1/8) of product of leukapheresis), we still obtained 9.7 × 10^6^ Tregs after sorting, which is sufficient for further clinical processing. Even if the fold increase in cell number is lower than 100 during the *ex-vivo* expansion, the number of isolated cells allows the process to achieve around 1 billion Tregs, which would be sufficient for clinical application with our target dose of 10 × 10^6^ Tregs per kg of body weight of the patient. Such Treg number is currently considered sufficient for testing in patients with autoimmune conditions as well as in transplantation for immuno tolerogenic effect and long-term graft survival [37].

In the next step, we introduced CD4 positive selection to obtain pre-enriched cells. In contrast to direct Treg sorting from leukocytes, our approach demands lower number of cells submitted to sorting. Our protocol is less time-consuming and requires lower number and amount of costly clinical grade antibodies (CD4, CD25, CD127) for sorting. In our approach, only one vial of clinical-grade CliniMACS^®^ CD4 reagent is used for CD4^+^ cells pre-enrichment step and one for subsequent sorting. Of note, since CD4 antibodies used for CliniMACS^®^ separation and for sorting are based on different clones, performing both procedures one after another is still efficient.

In our protocol, we decided to use FACS instead of immunomagnetic separation in order to obtain highly pure Treg population defined as CD4^+^CD25^high^CD127^−/lo^ cells. In contrast, immunomagnetic method includes during the selection process also T cells with intermediate or even low level of CD25 expression, which leads to contamination of the isolation product with also non-Treg cells. Therefore, protocols based only on immunomagnetic selection must use rapamycin during *in vitro* expansion to suppress proliferation of contaminating cells, which otherwise can overgrow the Treg population [34, 38, 39]. Rapamycin present in the culture media during expansion also suppresses Treg proliferation [26, 40], so more intensive stimulation with anti-CD3/CD28 beads is necessary, which can lead to cell exhaustion and expansion failure. Only FACS allows isolating cells expressing selectively high level of CD25 and obtaining highly purified Tregs without need for rapamycin. The disadvantage of FACS is that, so far there are no cGMP-compliant cell sorters widely available. Therefore, in order to meet cGMP requirements for clinical cell processing, we placed the BD FACSAriaIII sorter into the custom-made biosafety cabinet inside cGMP facility. We also performed built-in “preparation for aseptic sort procedure” to clean interior surfaces of the sorter, [41]. We also used gas-sterilized nozzle with sample line – the parts that are in contact with cells during sorting in order to eliminate the risk of contamination. Moreover for a quality control, before running cells for sorting, we sent a sample of sheath fluid for sterility testing to prove that the sorter is free of microbiological contaminants. FDA has already approved similar protocols for cleaning and maintenance under IND for example in Dr Tang/Bluestone's lab in UCSF (personal communication).

In our protocol, Tregs are submitted for *ex vivo* expansion for 13 days with two rounds of bead stimulation. In previous set of experiments, we set optimal time frame for Treg expansion as 13 days [6, 11, 26]. It allows generation of sufficient number of Tregs with well preserved phenotype stability and function [26].

We expanded Tregs after stimulation with anti-CD3/CD28 beads and in the presence of IL-2 in concentration of 1,000 UI/ml in a culture medium. We chose such concentration of IL-2 based on previously published protocols [11, 23, 26, 30, 42]. Moreover, it has been shown that higher concentration of IL-2 in the culture makes Tregs less prone to apoptosis caused by activation-induced cell death (AICD) [43]. During expansion both stimulatory pathways: via TCR/CD28 co-stimulation and IL-2 are shown to support the function and viability of Tregs [43]. Additionally, in our previous research-grade Treg expansions we also tested different concentrations of IL-2 (100; 1000; 10,000 UI/ml) and we found that concentration 1000 UI/ml provides the highest fold increase in Treg cell number with preservation of Treg viability and phenotype (unpublished data).

Tregs processed according to our protocol maintained their phenotype and function, which was confirmed at different time points of expansion and in *in vitro* suppression of proliferation assay. At the end of Treg processing anti-CD3/CD28 beads used for stimulation were removed from Treg cell product utilizing a clinical-grade magnet according to the previously published assay, which we validated in our laboratory [8, 44].

To characterize our final Treg product, we established appropriate release criteria according to the FDA requirements [29, 45] and previously approved Treg clinical trials in the USA [8–10]. It is essential that all quality and quantity checks for final product release criteria are performed in clinical, CAP- certified laboratories or utilizing properly validated own assays. For convenience, we sent out samples for sterility, % of CD4^+^, % of FoxP3^+^, % of CD8^+^ determination to the clinical hospital laboratories. We developed and validated own testing methods, only when otherwise unavailable. Our final Treg product met all the release criteria. We are aware of the shortcomings of these criteria; however, this is probably the best currently available panel, which can be analysed during the process of real-time product release. Whenever methodology allows to obtain the results within the timeframe of release, novel markers can be also included. For example, the analysis of the FOXP3-TSDR does not allow to include this trait as a part of the release criteria due to time-consuming processing of such analysis.

In summary, we described our Treg *ex vivo* expansion protocol successfully introducing leukapheresis as an alternative to blood drawing or UCB as the source of leukocytes. It provides higher initial number of Tregs and limits the risk of failure during the expansion phase. Having an excessive final number of Tregs not only allows the investigator to apply them instantly after expansion into patients, but also to cryopreserve a remaining dose of cells for later clinical use. This has a major practical advantage of opportunity for subsequent, repetitive dose as well as optimal timing of application. Additional introduction of CD4 positive selection limits number of cells and cost of sorting. In this study, we validated our Treg isolation and expansion protocol providing clinical-grade Treg product. After IND approval it can be further tested for safety and effectiveness in clinical trials as potential immunotherapy in cell/organ transplantation and immune disorders.

## MATERIALS AND METHODS

### Clinical-grade regulatory T cell isolation

**Figure 4:**
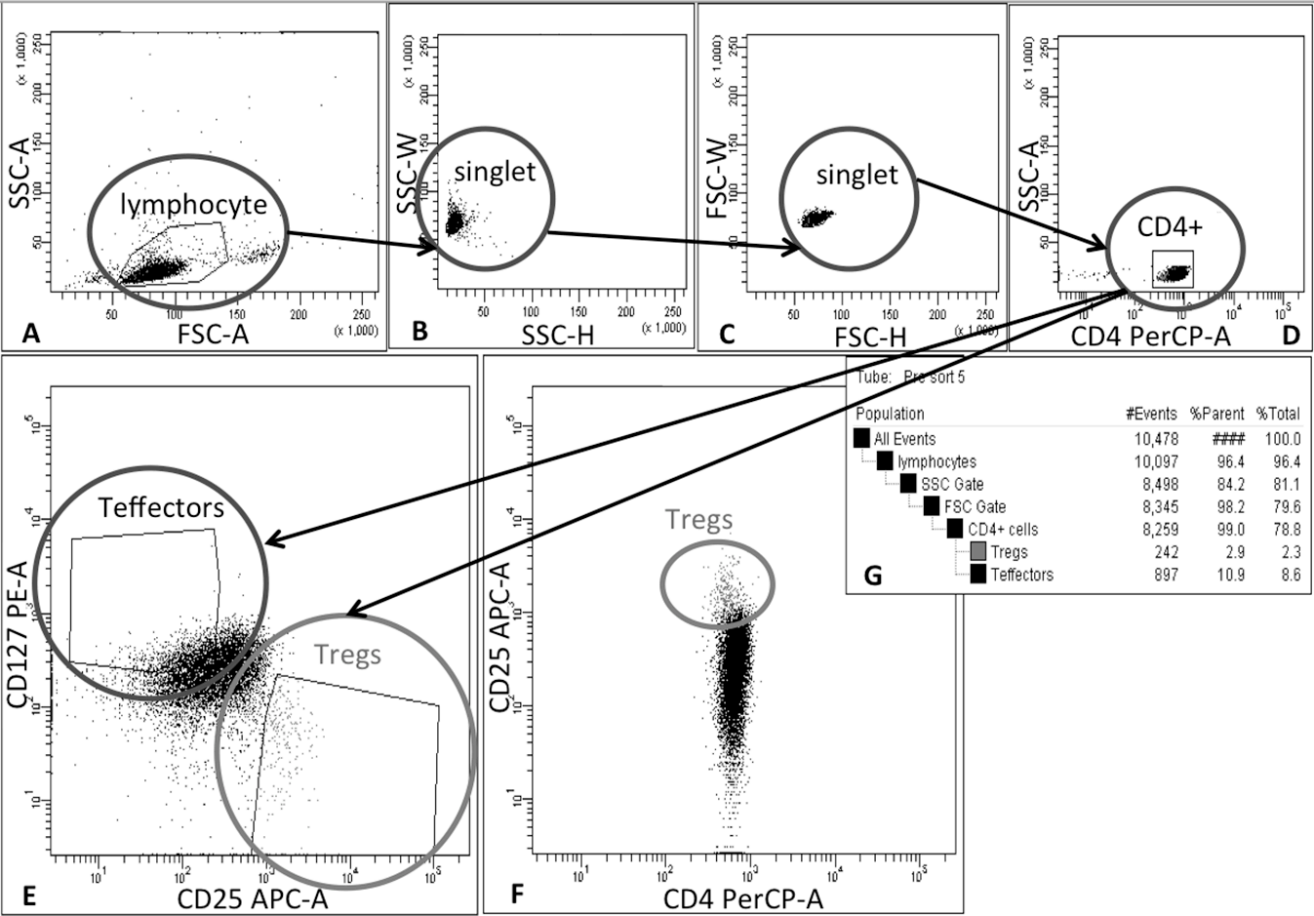
Schema of gating strategy for Treg sorting Tregs were isolated in 2-step process: firstly, CD4^+^ cells were pre-enriched from leukapheresis product via immunomagnetic positive selection on CliniMACS^®^ device and then cells were stained with monoclonal antibodies and separated by Fluorescence Activated Cell Sorting (FACS). During FACS cells were gated as follows: (**A**) lymphocytes were identified on forward (FSC) and side-scatter (SSC) plot, (**B**) and (**C**) doublets were excluded by applying SSC-H vs SSC-W and FSC-H vs FSC-W gates, (**D**) followed by CD4^+^ cell gating. (**E**) Finally, Tregs and Teffectors were gated as CD25^hi^CD127^lo/−^ and CD25^lo/−^CD127^+^ cells, respectively; (**F**) dot-plot CD4 PerCP vs CD25 APC was used as a reference for correct Treg gating based on lower CD4 expression in Treg population than in Teffectors. (**G**) table with population hierarchy and statistics generated by FACSDiva Software during cell sorting on FACSAria III cell sorter (BD Biosciences, San Jose, CA, USA

Tregs were isolated in a two-step process. In the first step, CD4^+^ cells were selected from leukapheresis product (AllCells, Alameda, CA, USA) obtained from healthy volunteers. In that process, immunomagnetic isolation on CliniMACS^®^ device (Miltenyi Biotec GmbH, Bergisch Gladbach, Germany) with clinical-grade reagents were utilized. The CD4^+^ cells isolation was performed without delay after receipt of leukapheresis product as suggested by the provider. CD4 isolation was performed on Miltenyi Biotec CliniMACS Instrument (V2.40) following user manual for cell preparation and magnetic labeling: after adding CliniMACS^®^ CD4 Reagent, the cell preparation bag was placed flat on an orbital rotator at room temperature for 30 minutes. After magnetic separation process, CD4^+^ cells were centrifuged, suspended in X-VIVO^™^ 20 medium (Lonza Walkersville, Inc., Walkersville, MD, USA) supplemented with 10% human AB serum (Valley Biomedical Products & Services, Inc., Winchester, VA, USA) and split into portions each containing 100 × 10^6^ cells. One of the portions was prepared directly for staining in 5 ml conical tube (BDBioscience, Bedford, MA, USA) and the rest of them put in the T-75 flasks (BDBiosciences, Bedford, MA, USA) and placed for short-term culture in a 37°C, 95% humidity, 5% CO_2_ incubator until the sorting of the first and subsequent portions was completed. Totally 5 × 10^8^ CD4^+^ cells were stained with clinical-grade fluorochrome-conjugated monoclonal antibodies: CD4 PerCP, CD25 APC, CD127 PE (BDBiosciences, San Jose, CA, USA). Next Tregs (CD4^+^CD25^hi^CD127^−^ cells) were sorted accordingly to the gating strategy shown on the Figure 4 with FACSAria III cell sorter (BDBiosciences, San Jose, CA, USA) adapted in a custom-designed biosafety cabinet (NuAire, Plymouth, MN, USA) inside our cGMP clean room facility class 10,000 utilizing gas-sterilized 70 μm nozzle and sample line. Sorting was performed with setting of “precision mode” and speed < 10,000 events/sec to sort highly pure Treg population. During the 48-hour sorting process, samples with collected Tregs were centrifuged, re-suspended *in vivo*^™^ 20 medium (Lonza Walkersville, Inc., Walkersville, MD, USA) supplemented with 10% human AB serum (Valley Biomedical Products & Services, Inc., Winchester, VA, USA) and 1000 U/ml of interleukin-2 (Proleukin^®^, Novartis Pharmaceuticals Canada Inc., Dorval, Quebec, Canada) - Treg Culture Medium and placed for a short-term culture in a 37°C, 95% humidity, 5% CO_2_ incubator. All collected samples during sorting were checked for the purity - % of CD4^+^CD25^hi^CD127^lo/−^ cells. During sorting, a portion of T effectors (CD4^+^CD25^lo^CD127^+^ cells) was also separated (Figure 4) at the same time for further Treg control assays.

### Clinical-grade Treg expansion

After sorting, Treg samples with the purity ≥ 95% (% of CD4^+^CD25^hi^CD127^lo/−^ cells) were pooled together, counted and placed in 96-well U-bottom tissue culture plates (BD Biosciences, Durham, NC, USA) at a concentration of 2 × 10^5^ cells per well suspended in Treg Culture Medium. For Treg stimulation MACS^®^ GMP ExpAct Treg Beads (Miltenyi Biotec GmbH, Bergisch Gladbach, Germany) at cell: bead ratio 1:1 were used. Tregs were cultured in a 37°C, 95% humidity, 5% CO_2_ incubator and were split with addition of fresh Treg Culture Medium every 24 - 48 hours to maintain the concentration of ~2 × 10^5^ cells per well. At day 7, Tregs were collected, counted and re-stimulated with fresh beads at the same ratio as for initiation of expansion.

**Figure 5:**
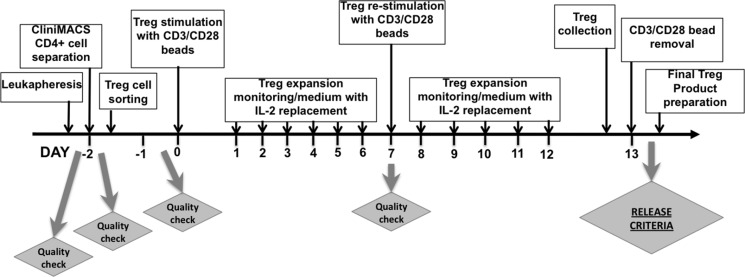
Timeline of Treg isolation and *ex-vivo* expansion The flowchart shows the procedure from the receipt of leukapheresis product, through CD4^+^ cells separation on CliniMACS^®^ device, Treg sorting (details of sorting strategy is described in Figure 4), Treg expansion, collection and preparation of Final Treg Product with indication of Quality Check Points during the process. During 48-hour sorting process, samples with sorted Tregs were centrifuged and collected, Tregs were re-suspended in Treg Culture Medium with IL-2 for short-term culture. After sorting, Tregs were pooled together, counted and distributed into 96-well U-bottom plates at concentration 2 × 10^5^ cells per well. Next, anti-CD3/CD28-coated beads were added and Tregs were expanded for 13 days. Treg culture was maintained on daily basis and Tregs were split with addition of fresh medium with IL-2. On day 7, Tregs from all wells were collected, sample of Tregs was retrieved for phenotype and viability analysis as Quality Check of expansion process. Then, Tregs were counted, re-distributed on plates and re-stimulated with fresh anti-CD3/CD28-coated beads. At day 13 of expansion, Tregs were collected, suspended in Final Treg Product Medium and anti-CD3/CD28 beads were removed on ClinExVivo^™^ MPC^®^ magnet. After bead removal, sample of Treg cell suspension was taken for Final Treg Product Testing.

On the 13th day, Tregs were collected, counted and suspended in the Final Treg Product Medium that contained 49.02% of Plasmalyte A (Baxter Healthcare Corporation, Deerfield, IL, USA), 49.02% of Dextrose, 5% and 0.45% NaCl (Baxter Healthcare Corporation, Deerfield, IL, USA), 1.96% of 25% Human Serum Albumin (Baxter Healthcare Corporation, Deerfield, IL, USA). Just after collection, MACS^®^ GMP ExpAct Treg Beads were removed on ClinExVivo^™^ MPC^®^ magnet (Life Technologies, Dynal Biotech ASA, Norway) in bags (Charter Medical, Ltd., Winston-Salem, NC, USA) connected together to create a closed system. After bead removal, 5 ml sample from Treg suspension was taken for a final Treg product count and testing. All procedures of Treg isolation and expansion were performed in cGMP clean room facility class 10,000. Samples for sterility testing were sent at subsequent steps of the procedure: from leukapheresis product, after CD4^+^ cells isolation, Treg sorting and after bead removal from Tregs. The schema of protocol for Treg isolation and expansion as timeline is shown in Figure 5.

### Quality control checks of Treg expansion

Just after Treg sorting- day 0, as well as at day 7 and 13, quality of Tregs were checked by FoxP3, CD4, CD25, CD127, CD45RA and CD62L expression analysis. Briefly, 2 × 10^5^ Tregs were harvested and processed according to manufacturer instructions of the FoxP3/Transcription Factor Staining Buffer Set (eBioscience, Inc., San Diego, CA, USA) and stained with the following antibodies: FoxP3 FITC (eBioscience, Inc., San Diego, CA, USA), CD4 PerCP, CD25 APC, CD127 PE, CD45RA PE-Cy7 (BDBiosciences, San Jose, CA, USA), CD62L APC-Alexa Fluor^®^ 750 (Invitrogen, Frederick, MD, USA). Samples were acquired on LSRFortessa^™^ Cell Analyser (BDBiosciences, San Jose, Cam, USA) and analyzed on FlowJo^®^ software (FlowJo, LLC., Ashland, OR, USA). Isotype and Fluorescence Minus One (FMO) controls were used for gate settings.

### Suppression of proliferation assay

To analyze Treg function after the expansion, we performed suppression of proliferation assay. Teffectors were stained with 5 μM carboxyfluorescein diacetate succinimidyl ester (CFSE) (Molecular Probes, Inc., Invitrogen, Eugene, OR, USA) and plated in concentration of 5 × 10^4^ cells per well on 96 well u-bottom plates in co-cultures with expanded Treg cells in different proportions (Treg:Teffector – 1:1, 1:2, 1:8). Cells were cultured in the presence of anti-CD3, anti-CD28-coated magnetic beads (LifeTechnologies, USA) at 1:1 bead to cell ratio and IL-2 at concentration 100 U/ml. Assay was prepared in triplicate. After 4 days of co-culture, CFSE dye dilution was measured using LSRFortessa^™^ Cell Analyser. Percentage of suppression was calculated in relation to 100% in condition of non-stimulated Teffectors.

### Release criteria of the final Treg product

The following Release Criteria were established based on FDA requirements and previously described clinical trials with Tregs conducted in USA: 1) sterility – to exclude presence of aerobic or anaerobic bacterial and fungal growth; 2) mycoplasma test (MycoAlert Mycoplasma Assay, Lonza Walkersville Inc., Walkersville, MD, USA) < 0.8; 3) endotoxin (EndoSafe^®^ PTS^™^ Endotoxin System, Charles River, Charleston, SC, USA) - < 5 EU/kg; 4) determination of residual expansion beads - < 100 per 3 × 10^6^ cell, 5) viability with 7 AminoActinomycin D (7AAD) - > 75%; 6) % of FoxP3^+^ cells - > 60%, 7) % of CD4^+^ cells - > 90%, 8) % of CD8^+^ cells - < 5%. Viability with 7AAD, % of FoxP3^+^, CD4^+^ and CD8^+^ cells were determined by flow cytometry. Most of the assays for release criteria were validated and performed by College of American Pathologists (CAP)- certified laboratories. Determination of residual expansion beads was performed “in house” after assay validation. Five ml of Treg suspension was retrieved from the infusion bag after beads removal and Tregs were tested to fulfill established Release Criteria.

### Pre-clinical grade Treg isolations and expansions

A non-clinical grade Treg isolations and expansions (*N* = 3) were performed in the same manner as described for clinical-grade procedures, but with use of non-clinical grade antibodies for Treg sorting: CD4PerCP-Cy^™^5.5, CD25APC, CD127 PE (BD Biosciences, San Jose, CA, USA). More than 1 × 10^6^ were sorted and they were expanded for 13 days as described above with monitoring of Treg stability by checking Treg markers (CD4, CD25, CD127, FoxP3) expression. Treg functionality was checked in suppression of proliferation assay at the end of expansion. After expansion, Tregs were collected and counted to assess fold increase in cell number.

### Ethical considerations

We obtained de-identified human cell leukapheresis product for the all experiments from the commercial vendor AllCells, Alameda, CA, USA, therefore the study was exempt from the Institute Review Board (IRB) review. In addition, vendor certified that the product was obtained from volunteers participating in an IRB or Human Subject Committee approved donor program and had current IRB approval. Vendor also certified that all donors provided informed consent for use of the cell for any research study.
